# Validating the regional estimates of changes in soil organic carbon by using the data from paired-sites: the case study of Mediterranean arable lands

**DOI:** 10.1186/s13021-021-00182-7

**Published:** 2021-06-07

**Authors:** Calogero Schillaci, Sergio Saia, Aldo Lipani, Alessia Perego, Claudio Zaccone, Marco Acutis

**Affiliations:** 1 Department of Agricultural and Environmental Science, University of Milan, 20133 Milan, Italy; 2 Department of Veterinary Sciences, University of Pisa, Via delle Piagge 2, 56129 Pisa, Italy; 3 Department of Civil, Environmental and Geomatic Engineering, University College London (UCL), Gower St, London, WC1E 6BT England; 4 Department of Biotechnologies, University of Verona, Strada Le Grazie 15, 37134 Verona, Italy

**Keywords:** Soil organic carbon (SOC), Soil monitoring, Minimum detectable change (MDC), Power analysis, Semi-arid

## Abstract

**Background:**

Legacy data are unique occasions for estimating soil organic carbon (SOC) concentration changes and spatial variability, but their use showed limitations due to the sampling schemes adopted and improvements may be needed in the analysis methodologies. When SOC changes is estimated with legacy data, the use of soil samples collected in different plots (i.e., non-paired data) may lead to biased results. In the present work, N = 302 georeferenced soil samples were selected from a regional (Sicily, south of Italy) soil database. An operational sampling approach was developed to spot SOC concentration changes from 1994 to 2017 in the same plots at the 0–30 cm soil depth and tested.

**Results:**

The measurements were conducted after computing the minimum number of samples needed to have a reliable estimate of SOC variation after 23 years. By applying an effect size based methodology, 30 out of 302 sites were resampled in 2017 to achieve a power of 80%, and an α = 0.05.

A Wilcoxon *test* applied to the variation of SOC from 1994 to 2017 suggested that there was not a statistical difference in SOC concentration after 23 years (Z = − 0.556; 2-tailed asymptotic significance = 0.578). In particular, only 40% of resampled sites showed a higher SOC concentration than in 2017.

**Conclusions:**

This finding contrasts with a previous SOC concentration increase that was found in 2008 (75.8% increase when estimated as differences of 2 models built with non-paired data), when compared to 1994 observed data (Z = − 9.119; 2-tailed asymptotic significance < 0.001).

This suggests that the use of legacy data to estimate SOC concentration dynamics requires soil resampling in the same locations to overcome the stochastic model errors. Further experiment is needed to identify the percentage of the sites to resample in order to align two legacy datasets in the same area.

## Background

Soil organic carbon (SOC) is a main contributor to fertility in agricultural soils, improving water accumulation and biodiversity [1]. Baseline SOC estimates and maps are generally built on legacy data [2], whereas any new soil samples collection in the same legacy locations are often scarce. Up to date SOC data and assessments are on the global agenda [3] and are necessary to evaluate many ecosystem characteristics such as resilience, productivity, ability of soil to provide a wide range of ecosystem services [4], and to gain precious insight into policy measures for soil preservation [5].

Prior to a SOC assessment, a sampling campaign would be needed, and the number of samples would affect obtained results. Many sampling size determination strategies have been proposed in the last decades to spot SOC changes [6–10]. Most of these have been suggested for new data acquisition and to overcome the problem of inaccessible lands. There is now a strong and growing need to utilize legacy soil data sources for monitoring SOC changes [11–13]. Regions that have soil monitoring networks need periodic recollection of soil samples to evaluate changes over time. Such resampling could be minimized to contain costs, but should be large enough to produce reliable estimates.

The need for long-term temporal paired sites is essential when aiming to depict SOC changes [14, 15]. Few countries have such monitoring schemes. The European Union (EU) started around 10 years ago with a Pan-European monitoring network to improve sustainable farming solutions and monitor soil pollution [16, 17].

Soil and SOC conservation is critically important in semi-arid areas, where desertification risk is increasing [18]. For these areas, a recent increase in the output of literature regarding SOC accounting and spatial modelling [19], legacy databases [20], and digital soil mapping [21] has been noted.

Legacy soil data and soil maps could be integrated into a unified database. This would provide special insight into hard-to-sample areas, past and present trends, and insight into the application of proper modelling procedures.

Recently, the development of digital soil mapping and pedometrics, associated with the presence of an ample archive of historical soil data has allowed for the assessment of SOC patterns at a country scale with relatively high accuracy [22, 23]. However, models can amplify uncertainty when the assessment is based on multiple predictors [24, 25].

In some areas of the world, a lack of recent SOC measurements is prompting a rediscovery of legacy data which is in the process of being fully integrated into mapping methods at an operational level [26].

SOC distribution is determined by multiple factors [27], the importance of which vary mainly with bioclimatic conditions. It is therefore hard to delineate general functions that explain the world SOC distribution using only geographical positions, although a general inverse correlation was found with average annual air temperature on a regional scale between 52° N and 40° S and a direct correlation beyond this region [28].

Land use and land use change is also a main driver of SOC stocks, although mechanisms of SOC dynamics seem to be often independent of the ecosystem type or land use [29]. Using a meta-analytic approach, Guo and Gifford [30] showed that around 50% of SOC is gained in the transition from cropland to secondary vegetation communities, and recent papers confirmed such a trend [31, 32].

Sommer and Bossio [34] hypothesized that SOC sequestration in arable land can show a 0.012–0.027% annual increase in the first two decades after the establishment of SOC preservation practices, after which a saturation occurs and the increase ceases. Following the same hypothesis, Zomer et al. [34] presented a global assessment of cropland SOC under the aforementioned scenarios and found that the potential SOC sequestration in cropland is below 53% of the 4p1000 target [35].

High spatial variability and temporal trends induced spatial modellers to design reliable sampling strategies [9, 36, 37] and develop efficient methods to compare intra-field and inter-field variations [38] with similar agro-ecological conditions over the course of two decades [39]. Application of this technique was carried out to determine the effects of sampling density on interpolation accuracy [40] and uncertainty assessment [41, 42].

Cropland covers 12.6% of the world’s surface (FAOSTAT data, accessed in 2019). Cropland SOC content has been mapped on a global scale using the WoSiS database [34, 43, 44] and SOC maps were obtained by applying Generalized Additive Models (GAM) and machine learning methods.

At the European level, cropland plays an important role. Due to the large area covered, cropland acts as a potential carbon (C) sink. If considering a biomass return of up to 45 Mg C per year in raw organic materials, the biological potential of cropland for C storage is on the order of 90–120 Mg C per year [45, 46]. In particular, Smith [46] demonstrated that models of SOC changes should be used with statistical power analyses for planning sample design to determine density and time of sampling during experiments.

Little is known about long-term SOC changes in Mediterranean semi-arid arable lands, which are frequently dominated by winter-growing species (mostly cereals and legumes) in rotation with fallow periods characterized by various crop residue management practices. Field crop production in these areas can cause SOC depletion [47] and soil loss by erosion, especially when conventional tillage (e.g., ploughing) is continuously applied. Conversely, no-till has been shown to be strongly beneficial compared to conventional tillage in semi-arid climates with an aridity index lower than 0.52 ± 0.03 [48]. Land management practices such as reduction of tillage intensity [49], addition of manure and sowing cover crops could help to increase SOC contents and keep significant amounts of nutrients [50, 51].

In addition, in Mediterranean areas, frequent fire events can burn tons of biomass; this lowers the yearly C input derived from crop residues utilization while increasing SOC permanence, and affect the cycle of several nutrients including nitrogen [52]. In these areas, short-term SOC changes due to management practices (and especially land use) can temporarily override background changes [53] since length of cultivation is a main driver of SOC variation [54]. Such information can allow for the determination of a minimum sample size to test a hypothesis effectively. Defining the sample size and location is required to enhance the power analysis while reducing laboratory costs and maximizing the accuracy of the assessment [55].

This experiment aimed at verifying whether or not a legacy estimation of SOC changes (1994–2008 modelresults) from non-paired data [56] matches the SOC variation measured in paired sites after 23 years (1994–2017).

Thirty temporal paired sites from Sicily (South of Italy) and under continuous crop cover were resampled [20] and included in the present study. The analysis focused on arable land as it represents the main land use in the study area. The land cover of these sites was verified using historical remote sensing imagery to confirm that each site was continuously cultivated during the intervening period. Minimum sample size was determined and locations were randomly selected. Topsoil SOC contents were determined using the same laboratory method as in 1994 and 2008 (Walkley and Black, 1934).

## Methods

### Study area

Sicily (25,286 km^2^) is the largest Mediterranean island, belong to the semi-arid to arid climates, characterised by prolonged droughts from mid or late spring to early or mid-fall, with high energy storms in fall and winter.

Sicily has an highly variable geomorphological setting, resulting from sedimentary processes, tectonics, climatic changes, and human activity [58]. Fantappiè et al. [58] classified the geomorphological macro-areas into five typical assemblage of landforms and geomorphological processes: (i) volcanic landscape, craters, lava flows, and volcanic ash fields, the slopes of these areas are commonly characterized by anthropogenic terraces, used for viticulture, orchards, and fruit trees; (ii) coastline; (iii) hyblean platform: in the south-eastern part of the region which is a carbonate plateau between 450 and 600 m, characterized by many fluvial valleys and karst features; (iv) calcareous mountains and hills mainly in the southern coast and Inland hills characterized by clay deposits and flysch formations; (v) steep slopes ridges of Nebrodi and Peloritan mountains which are mainly hills formed on arenites and metamorphic rocks.

Soil systems were derived from the ‘Soil map of Italy’ [59, 60]. Soils of the north-eastern part, developed on igneous and metamorphic rocks and are mainly Cambisols and Leptosols. The Etna volcano influenced a large part (30 km radius) of the island; soils of this territory are mainly Leptosols, Cambisols, Regosols and Andosols, whereas towards the south of the Volcano a fluvial alluvial until the coastal plains of are mainly Cambisols, Calcisols, Luvisols and Vertisols. Northern coastal and alluvial plains soils developed on tertiary calcareous rocks and sediments originated Cambisols, Vertisols and Luvisols. A different soil series can be observed on the hills and mountains on lime-stone and igneous rocks of south east Sicily (Hyblean plateau) with Cambisols, Leptosols and Andosols. From the south coast to the west coastal and hilly lands, the soils developed from clayey flysch, limestone, sandstone, gypsum and coastal plains and are mainly Luvisols, Vertisols and Regosols with high carbonates content.

Rainfed arable land selected using the CORINE code 211 is the most common land cover class in the area under study with roughly 300,000 ha yearly under cultivation with durum wheat [61].

Rainfed arable lands represented the target land cover in the study area as they represent 60% of the surface in this region. Thus, it is a primary candidate for C sequestration and mitigation of the anthropogenic impact on the landscape [62, 63]. The land is predominantly under private ownership and the average farm size is around 6 ha, in general family-run businesses (all farm types), with approximately 10% of foreign labour [61].

### Sampling campaign

In the rainfed arable lands of Sicily, the seedbed is generally prepared by soil ploughing during late summer and one or two harrowing in early fall. The amount of nitrogen (N) applied in non-legume field crops is usually between 80 and 100 kg ha^−1^ year^−1^ and durum wheat yield is between 2 and 4 Mg ha^−1^ (with a harvest index ranging from 45 to 55%, and therefore with a similar straw yield). Land cover data was derived from aerial imagery found in the Geographic information system of Sicily (SITR; http://www.sitr.regione.sicilia.it/).

### Expected SOC difference assumptions and sampling size determination

Data obtained from a sampling campaign carried out in 1993–1994 were used in the present work. In particular, data about the rainfed arable land (CORINE code 211) were identified using the optimization procedure shown in Schillaci et al. (2019). Briefly, the whole legacy database consists of 6674 checked samples. Samples falling into the CORINE Land use 2 (agricultural lands which comprises, rainfed and irrigated arable soils, olive grove, vineyards and fruit trees cultivation) were 5471 (pedological profiles) from 2886 locations. Within these 2886 locations, samples from the sole CORINE Land use 2.1 (rainfed arable land) were 2162 from 880 locations.

**Fig.1:**
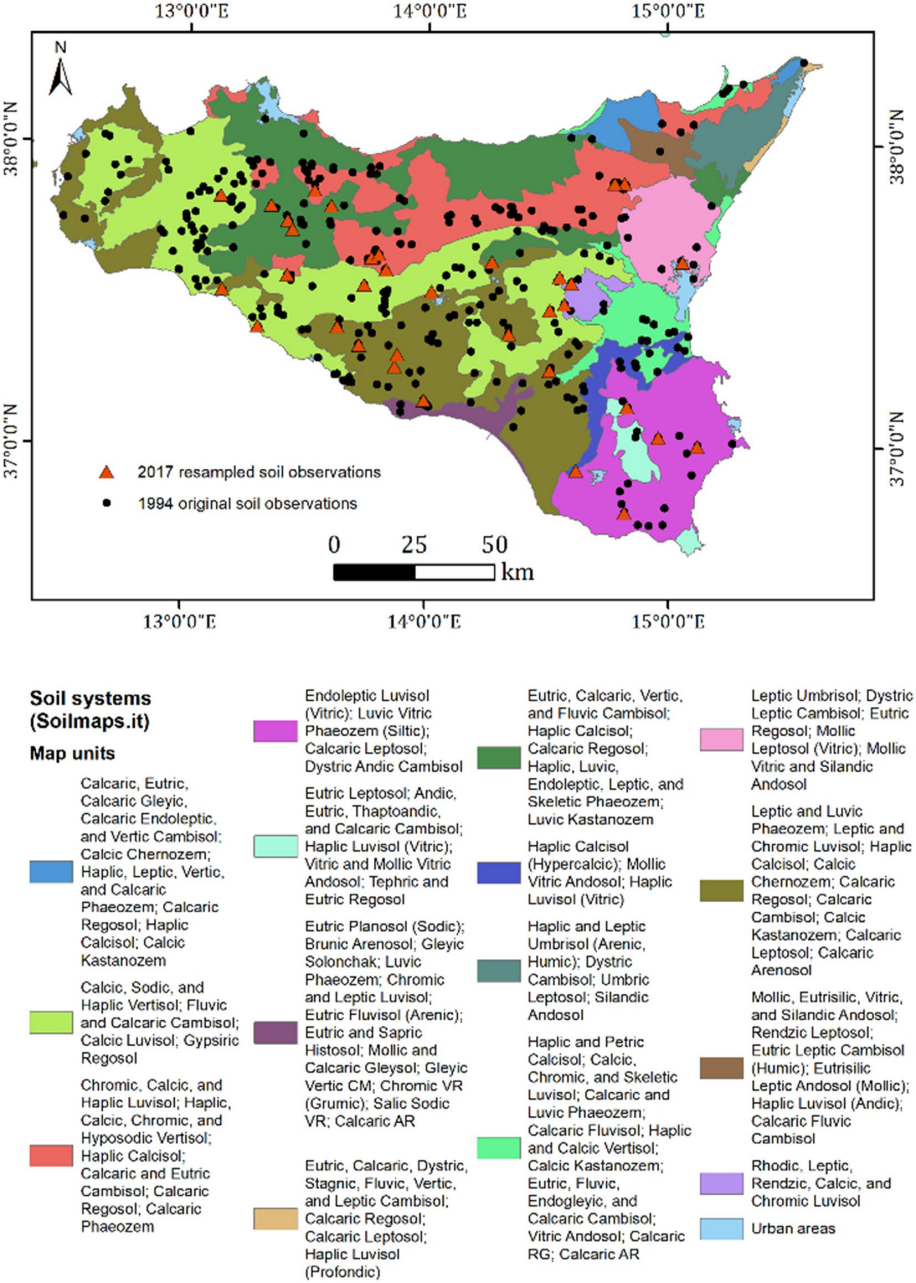
Study area and main soil systems from the Italian soil information system http://www.soilmaps.it/. The samples collected only in 1994 (black dots) and both in 1994 and 2017 (red triangles) were projected on the map

A *power analysis* [64] was used to find the minimum number of samples needed to determine the SOC change with time (from 1994 to 2017). The expected change with time was derived from a modelled SOC variation (1994 to 2008) at the regional level, as observed in Schillaci et al. [56]. In particular, samples from the same soil layer, land use and sub-area were used (Fig. 1).

To define the effect size, means and standard deviation of the oldest SOC survey (1993) and the hypothesized change after 15 years (2008) were used. The effect size was computed at different degree of confidence (0.1, 0.05, 0.01) using the G-Power software [65]. The estimated change comes from the difference between the average topsoil SOC concentration (0–30 cm) measured in 1994 and the estimated values at the same location in 2008 (predicted) at a 1-km spatial scale [56]. This allows for six sets of data (Table 1) used as raw or log_10_ transformed data, none of which was normally distributed after a Shapiro–Wilk test of normality (Table 2).

**Table 1:** Datasets use in the present experiment

Abbreviation	Number of sites	Description
LEG-SOC94	302	Data of measured topsoil SOC collected in the 1994 in land use CORINE 2.1 also used in [56] after a normalization procedure for depth. See further for explanation of the normalization process
EST-SOC08	302	Data of estimated topsoil SOC in land use CORINE 2.1 extracted in the coinciding location of the LEG-SOC94 after the BRT modelling built with samples taken in 2008 and provided in [56]
SOC94	30	Random samples from the LEG-SOC94 in land use CORINE 2.1 after control for stability of land use until the 2017. See further for explanation of the strategy to establish the number of samples and location
SOC17	30	Samples taken in April 2017 in coinciding locations of the C94
EST-SOC08- LEG-SOC94	302	Differences between data estimated in 2008 in the same locations of data collected in 1994
SOC17- SOC94	30	Differences between data measured in 2017 in the same locations of data selected in 1994

log_10_ of each of these databases were also computed

**Table 2:** Results of the Shapiro–Wilk for the raw and log- transformed data of the 1994, 2008, and 2017

Shapiro–Wilk	Stat	Sign
Raw—LEG-SOC94^a^	0.903	< 0.001
Raw—EST-SOC08^a^	0.798	< 0.001
log-LEG-SOC94^a^	0.980	< 0.001
log-EST-SOC08^a^	0.883	< 0.001
Differences of raw data (EST-SOC08-LEG-SOC94)^a^	0.961	< 0.001
Differences of log data (EST-SOC08-LEG-SOC94)^a^	0.960	< 0.001
Raw—SOC94^b^	0.833	< 0.001
Raw—SOC17^b^	0.809	< 0.001
log—SOC94^b^	**0.951**	**0.183**
log—SOC17^b^	**0.965**	**0.404**
Differences of raw data (SOC17-SOC94)^b^	0.886	0.004
Differences of log data (SOC17-SOC94)^b^	0.824	< 0.001

According to the Shapiro-Wilk test, chosen the alpha level 0.05 and the p-value is less than 0.05, the null hypothesis that the data are normally distributed is rejected. If the p-value is greater than 0.05, then the null hypothesis is not rejected. Values in bold indicated normality

Please see Table 1 for a description of each dataset

^a^degrees of freedom = 302

^b^degrees of freedom = 30

The *expected difference* between 1994 (measured data in LEG-SOC94) and 2008 (estimated data in EST-SOC08) was subjected to the effect size computation (i.e., Cohen’s *d*). Standard deviation of legacy data was computed. Cohen’s *d* provided the minimum number of sites to be sampled in the 2017 survey on the original locations of the 1994 (LEG-SOC94) to ascertain if a change in SOC content occurred. When this number was achieved, the sampling consisted in the collection of topsoil (0–30 cm) in 30 randomly selected sites for which coordinates were reported with up to six digit precision (thus with an error < 30 m; Schillaci et al. 2019). This random selection was performed within the set of locations were SOC was determined in 1994 (SOC94).

A *two-tailed paired* Wilcoxon*-test* was performed using IBM SPSS software 26 [66], because, despite previous findings [56], there was uncertainty regarding the pattern of differences between SOC17 and SOC94. Such uncertainty derived from the internal variability of the model used in Schillaci et al. (2017) suffered by the absence of paired locations. Indeed, dynamics of the difference from the data in 1994 (measured in LEG-SOC94) to 2008 (estimated in EST-SOC08) was on average positive for each land use, but with a high variation.

### Calculations of the legacy topsoil SOC

Original legacy SOC data were collected at various depths. To compare SOC data sampled in the 2017 at a uniform depth to the ones sampled in 1993, the Hobley and Wilson (2016) method was used to uniform the SOC concentration of the former sampling campaign. Such a method is based on an exponential generalized function, as follows:

1 $$SOC\left( d \right){\text{ }} = {\text{ }}SO{C_0} \times {e^ - }^{k \times d}$$

where *d* is the depth (expressed in meters), *SOC*_*0*_ is the concentration of SOC at the soil surface (%), and *k* is the depletion constant (*m*^*−1*^). The Hobley and Wilson (2016) method first fits this model and finds an optimal *k* and *SOC*_*0*_for each location, then computes SOC at any depth (*d*). Note that in the original work [67], Eq. (1) also contains an additional term (i.e., SOC∞) modelling the concentration of residual SOC to a soil depth tending to infinity. In this study, SOC∞ was assumed to be null.

In this work, the Hobley and Wilson (2016) method was applied as follows: in location (10 locations) where > 2 layers were sampled, the model was directly fitted applying a SOC_(0.30 m)_ depth threshold. For those locations where the number of layers sampled was ≤ 2 (292 locations) the function could not be fit. In this case, the above model was first fit on all locations in order to find an expected *SOC*_*0*_ and *k* values. This resulted in *k* = 0.4815 and *SOC*_*0*_ = 1.4396. Then, using only the *k* found, *SOC*_*0*_was computed for each location using Eq. (1) as follows:

2 $$SO{C_{0 = }}SOC\left( {d'} \right) \times {\left[ {{e^{ - 0.4815 \times d'}}} \right]^{ - 1}}$$

where ${d'}$ is the depth of one data point of the locations for which SOC concentrations are known. The accuracy of the fitted depth functions was expressed with the Root Mean Square Error (RMSE) and the Mean Absolute Error (MAE).

### Historical land use and soil type in resampled sites

**Fig. 2:**
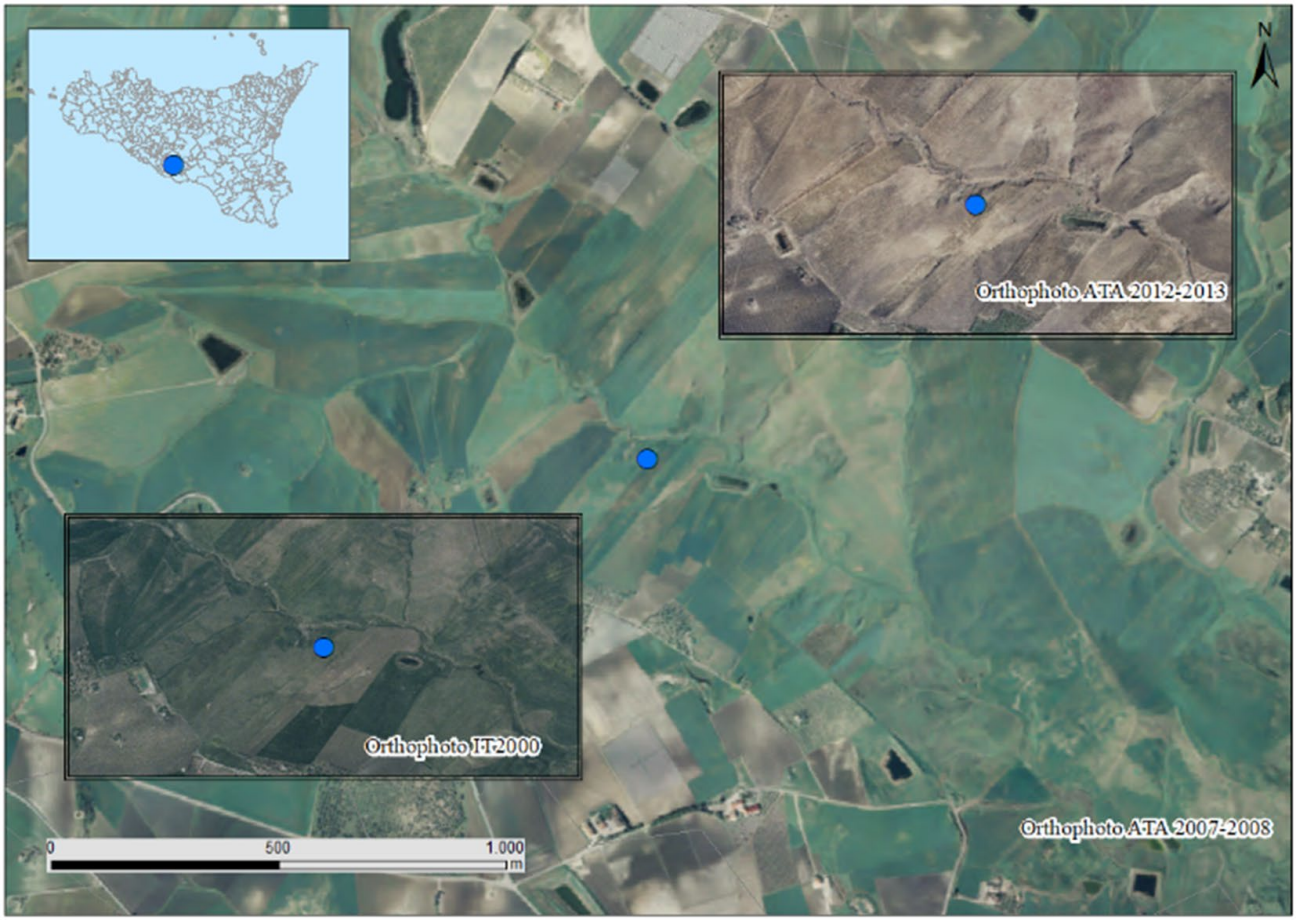
Example of checking of the actual land use along the course from the 1994 to the 2017 in a sampling location by means of a visual interpretation of the Orthophoto taken from a Web Mapping Services (WMS)

Web Mapping Services (WMS) from the regional geodata service (http://www.sitr.regione.sicilia.it/), consisting of aerial photograph surveys, were used to check the historical land cover. Sites were checked to ascertain that these sites maintained the same land use during the intervening period. To ensure this, aerial photographs of at least 3 surveys carried out from 1994 to 2017 (Fig. 2) were consulted. To avoid sites being temporarily converted to short-term grassland or bare soil or abandoned, local farmers were also interviewed.

Soil samples from the selected sites, rainfed arable land (N = 302) derived from the 1994 sampling campaign, and the resampled soils (N = 30) had a consistent distribution among the main soil systems according with the Soil map of Italy (59) (Fig. 1). Around 80% of sites were Vertisols or Cambisols or Regosols according with the World Reference Based WRB [68]. All of these soils frequently showed calcic or calcaric subgroups.

### Soil organic carbon determination

SOC17 measurements were carried out using the Walkley–Black method [69], i.e., a chromic acid wet oxidation method. Briefly, oxidizable organic C in the soil is oxidized by 0.167 mol L^−1^ potassium dichromate (K_2_Cr_2_O_7_) solution in concentrated sulphuric acid (H_2_SO_4_). The excess dichromate in the extract was then titrated with 0.5 mol L^−1^ iron (II) sulfate heptahydrate (FeSO_4_ 7H_2_O). The heat of reaction raises the temperature which is sufficient to induce substantial oxidation. The SOC values so obtained were within the range of LEG-SOC94. In LEG-SOC94, soil data were georeferenced, and this has allowed for the resampling at exact (field site) location.

### Computation of SOC differences between 1994 and 2017 by means of ad hoc sampling

Since the residues from the means of the logarithm_10_ of SOC94 and SOC17 were normally distributed (Table 2), the log_10_-SOC variation from 1994 to 2017 was tested for difference from zero by means of a paired *t*–test [70]. This does not assume that observations within each group are normal, but only that their residuals are normal [71, 72] (Table 2). Such assumptions were only partly met, since the log distribution were normal, whereas the differences between log were not normal but its skewness was comprised between − 1.96 and  + 1.96. To overcome such problems, a bootstrap ANOVA was performed [73]. Changes in cropland SOC content have previously been accounted for with the same statistical approach [74] where no subsampling was carried out to assess the SOC change.

### Results

#### Power analysis

Farmer interviews confirmed that the land use of sampling sites did not change, i.e., they have been continuously cultivated during the intervening period. The crop rotation generally consisted of durum wheat (*Triticum durum* Desf.) followed by broad beans (*Vicia faba* L.), or other pulses alternated with fallow. The sampling campaign LEG-SOC94 involved 302 sites, with a SOC concentration in the CORINE land use 2.1 (arable) of 1.01 ± 0.59% (mean ± s.d.; Table 3) after normalization to 0.3 m depth.

**Table 3:** Descriptive statistics and main quantiles of each of the dataset used in the present study

	SOC94	SOC17	log of SOC94	log of SOC17	LEG-SOC94 (measured)	EST-SOC08 (estimated)^b^	log of LEG-SOC94 (measured)	log of EST-SOC08 (estimated)^b^
Mean^a^	1.31	1.23	0.00550	0.03800	1.01	1.38	− 0.06983	0.12939
Standard deviation	0.96	0.67	0.33708	0.20808	0.59	0.39	0.26742	0.10394
Variance	0.93	0.45	0.11362	0.04330	0.35	0.15	0.07151	0.01080
Kurtosis	3.55	3.28	1.22	0.52	4.40	3.10	0.57	0.96
Skewness	1.76	1.78	− 0.69	0.36	1.53	1.79	− 0.55	1.23
n	30	30	30	30	302	302	302	302
Quantiles								
Min	0.120	0.400	− 0.9208	− 0.3979	0.100	0.953	− 1.000	− 0.021
0.010	0.149	0.429	− 0.8445	− 0.3698	0.150	1.001	− 0.824	0.000
0.025	0.193	0.473	− 0.7300	− 0.3277	0.215	1.012	− 0.667	0.005
0.050	0.270	0.545	− 0.5783	− 0.2654	0.300	1.027	− 0.523	0.012
0.250	0.780	0.803	− 0.1079	− 0.0956	0.560	1.142	− 0.252	0.058
0.500	1.050	1.100	0.0205	0.0414	0.945	1.253	− 0.025	0.098
0.750	1.633	1.353	0.2128	0.1310	1.328	1.487	0.123	0.172
0.950	3.343	2.830	0.5240	0.4493	2.099	2.219	0.322	0.346
0.975	3.699	3.128	0.5648	0.4952	2.260	2.477	0.354	0.394
0.990	4.156	3.171	0.6155	0.5012	2.820	2.773	0.450	0.443
Max	4.460	3.200	0.6493	0.5051	4.330	2.969	0.636	0.473

^a^for logs, mean of log represent the arithmetic mean of the logarithms_10_ of the SOC values expressed in %

^b^such an estimation is from the 302 sites in which a measure was available 1994 and applying the 1-km resolution estimation process used in the 2008 described in [56]

This value was significantly lower than the mean SOC in the 30 sites selected in 1994 (i.e., the SOC94, 1.31%; Table 3), but very close to its median (1.05%). Mean (predicted) SOC of the EST-SOC08 database was 1.38 ± 0.39% (mean ± s.d., with a median of 1.25%). SOC was expected to vary in the cropland between the 1994 and the 2008 values when using original data or log_10_ data (Table 4). Such variation was due to an increase in SOC in 75.8% of the sites (2008). The SOC calculated effect size was 0.54 for the original data and 0.69 for the log_10_ data (Table 5). According to Cohen [75] such effect sizes correspond to ‘*medium to high’* effect, which needed a minimum sample size ranging from 15 to 45 samples to be able to detect a SOC variation.

**Table 4:** Wilcoxon test for the difference between the soil organic carbon (SOC) of the arable lands (CORINE 2.1) in the 0–30 cm layer in 1994 (LEG-SOC94) and estimated SOC in 2008 (EST-SOC08) in the coinciding locations of the samples taken in 1994

		Raw		log	
Ranks	N	Mean ranks	Sum of ranks	Mean ranks	Sum of ranks
Negative ranks (EST-SOC08 < LEG-SOC94)	73	123.6	9026	100.3	7320
Positive ranks (EST-SOC08 > LEG-SOC94)	229	160.4	36,727	167.8	38,433
Ties (EST-SOC08 = LEG-SOC94)	0				
Test statistics based on positive ranks (Wilcoxon)					
Z based on negative ranks		− 9.119		− 10.243	
Asymptotic significance (2-tailed)		< 0.001		< 0.001

**Table 5:** Output parameters of the a priori power analysis computation process at varying the α (0.10; 0.05; or 0.01)

*α*	*0.10*	*0.05*	*0.01*
Raw data			
Noncentrality parameter δ	2.6008000	2.9077828	3.5612921
Critical t	1.7174255	2.0495831	2.6981518
Degrees of freedom	21.9183118	27.6478898	41.9718346
Minimum sample size needed	24	30	45
Actual power	0.8090485	0.8010923	0.8005742
log_10_ data			
Noncentrality parameter δ	2.6384055	3.0465682	3.6685540
Critical t	1.7676466	2.1001020	2.7730806
Degrees of freedom	13.3239449	18.0985932	26.6929601
Minimum sample size needed	15	20	29
Actual power	0.8040250	0.8216418	0.8056966

The process was carried out through the G-Power software with the Wilcoxon test for non-normal distributed datasets [65]. Input data were from 302 measured samples of SOC in 1994 and modelled SOC in 2008, each of which expressed as either raw or log10 data. Minimum power was set to 0.80

Given this effect size and the power chosen for the Wilcoxon test, which is by default set to 80%, and a significance level of 5%, the calculated sample size required would be 30 samples. Such a value could sound quite small; the reason is the huge difference expected, i.e., when a smaller difference need to be spotted a larger number of samples will be needed. These were identified in LEG-SOC94 and collected in their respective paired locations SOC17.

In 2017, only 12 sites showing a SOC concentration higher than 1994 were found, so SOC variation from 1994 to 2017 depended more on the SOC difference within each pair of samples than on the % of samples in 2017 having a SOC higher than in 1994.

### Descriptive statistics of SOC and SOC variation from 1994 to 2017

**Fig. 3:**
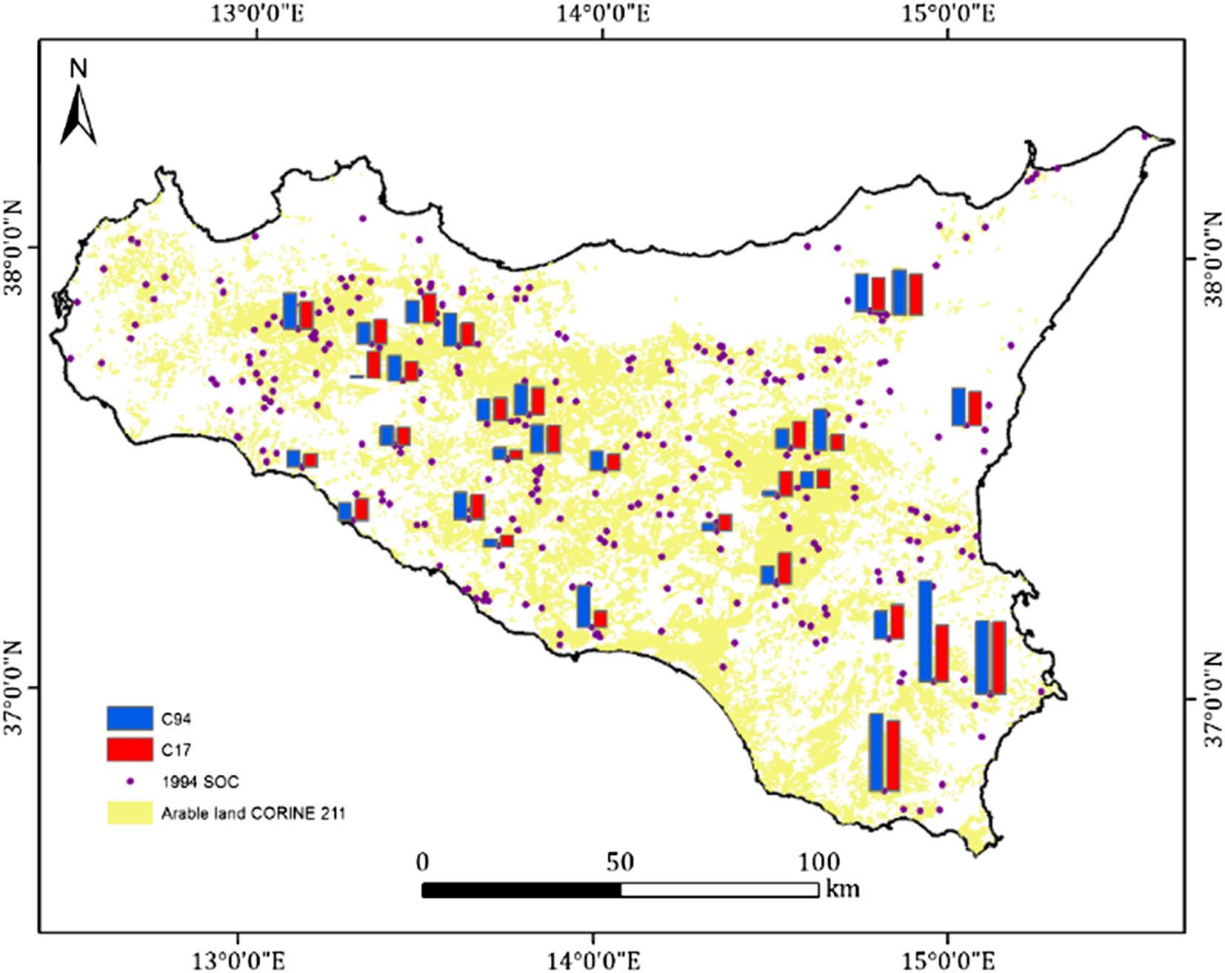
Study area, original soil data (dots) and original compared to new soil samples (histograms, background mask the Arable land cover from CORINE 2000, code 211 (yellow). Blue bars for SOC94, red bars for SOC17

**Fig. 4:**
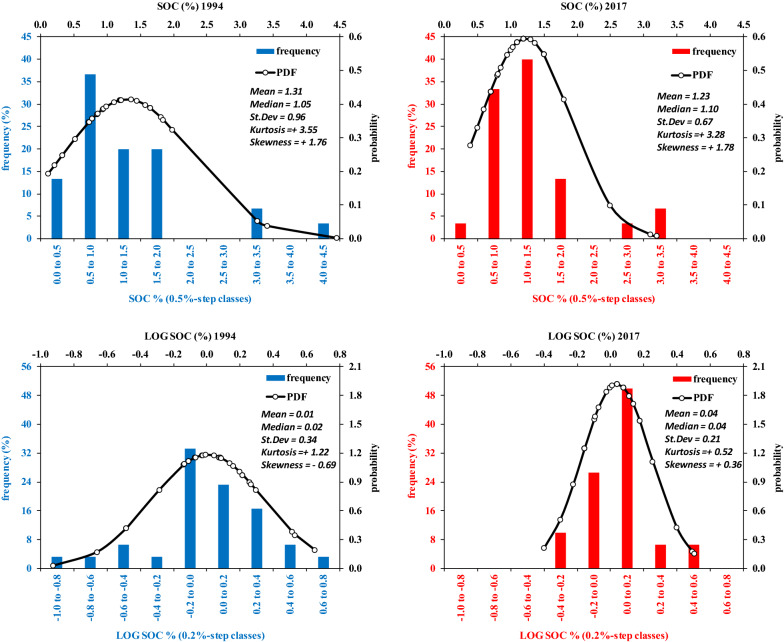
Distribution of the 30 samples of SOC 94 (blue bars) and SOC17 (red bars) and relative probability distribution function. Upper panels are for raw data, lower panels for log

**Fig. 5:**
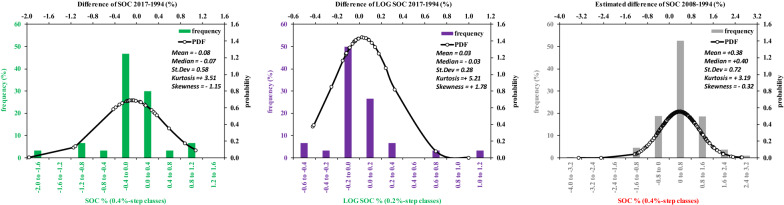
Distribution of the difference between pairs of the 30 samples of SOC17-SOC94 (expressed as raw data, green bars; or their logs, purple bars) and relative probability distribution function. Differences between the 302 original data (LEG-SOC94) and the 302 estimated data of 2008 (EST-SOC08) is also shown (grey bars). Positive values indicate an increase in SOC with time

SOC distribution in the datasets used in the present experiment had different means and standard deviations, but similar skewness and kurtosis (Table 3). SOC94 had a mean of 1.31 ± 0.96% (mean ± s.d.), whereas in the LEG-SOC94 sites, mean was 1.01 ± 0.59% (Fig. 3). The SOC17 mean was on average significantly lower than EST-SOC08, but with similar distribution properties to both the SOC94 and LEG-SOC94. Also, the SOC17 mean was slightly lower (− 6.1% relative change, − 0.08% absolute change) than the mean of SOC94, but not than the EST-SOC08. The accuracy of the fitted depth functions was expressed with the RMSE and MAE resulting in 0.36 and 0.23 respectively. Transformation to logarithms improved the distribution properties in term of skewness and kurtosis, especially for SOC17 (Fig. 4). According to the Wilcoxon test, the change in SOC between SOC94 and SOC17 did not appear different from zero (Table 6 and Fig. 5). We underline that different sub-samples can bring to similar results but regional patterns of SOC may influence the sub-sample mean.

**Table 6:** Results of the Wilcoxon tests of the mean difference of 30 samples of soil organic carbon measured in the 2017 (SOC17) and 1994 (SOC94) as difference of the raw data or log-transformed

Ranks	Raw			logs		
	N	Mean ranks	Sum of ranks	N	Mean ranks	Sum of ranks
Negative ranks (SOC17 < SOC94)	18	14.42	259.5	16	12.75	204
Positive ranks (SOC17 > SOC94)	12	17.13	205.5	12	16.83	202
Ties (SOC17 = SOC94)	0			2		
Test statistics based on positive ranks (Wilcoxon)						
Z based on negative ranks	− 0.556			− 0.023		
Asymptotic significance (2-tailed)	0.578			0.982		

Positive mean indicates a mean increase in SOC with time

The t-test applied to the log_10_ of SOC17 and SOC94, in which residues were normally distributed, provided similar results compared to the Wilcoxon test carried out on the same data (SOC difference =  + 0.03300 ± 0.27659, mean ± s.d.; C.I_95%_  − 0.07028 to + 0.13628; t =  + 0.653; 2-tails significance = 0.52). The bootstrap ANOVA also provided consistent results compared to the Wilcoxon test (Table 7), but notably, the difference in the log-SOC between the sampling campaigns had 95% confidence intervals marginally overlapping zero.

**Table 7:** Results of the bootstrap ANOVA on the differences between each pair of dataset

s	Bias	Std. Error	Sig. (2-tailed)	Lower 95% C.I.^a^	Upper 95% C.I
Differences of raw data (SOC17-SOC94)	0.0013	0.1049	0.4780	− 0.2923	0.1167
Differences of log data (SOC17-SOC94)	0.0007	0.0505	0.5400	− 0.0583	0.1394

Bootstrap samples were 10,000

N = 30

^a^*C.I.* confidence interval

### Discussion

This work was aimed at assessing the reliability of estimations of topsoil SOC changes with non-paired data using time-paired sampling. According to our results, the previous regional SOC estimates must be reconsidered as almost unchanged for rainfed arable, and the expected increase found in 40% of the sites after the 23-year timeframe can be traced back to the lower fire occurrence and the sediment redistribution.

This findings can be considered more accurate compared to the previous estimate [56] thanks to the use of temporal paired-sites instead of estimates based on different locations. Locations were further checked for land use continuity. The previous analyses based on modelled data over a 15-year span (from 1994 to 2008) predicted a mean relative increase of around the 21% of SOC content in arable lands, but such an increase was affected by a strong variability in the real plot scale land use when survey data was reviewed. By using a monitoring network spanning 30 years, Gubler et al. [76] found that SOC dynamic is more determined by a change in land use than other predictors in a colder climate (Switzerland) agro-ecosystem.

According to a hypothetical linear growth, the increase expected from 1994 to 2017, which was the target period of the present study, was predicted to be a relative SOC increase of around 30%. In this study, no evidence of this magnitude of increase was found, and no such difference was seen in neither the original data nor in the log transformed data. When using a similar amount of data from meadows, Gubler et al. [76] found that the minimum detectable change in 10 to 100 years (at a power of the 80%, such as in the present study) spanned from approximately 2 to 6%, respectively. Bellamy et al. [15] showed that variation in time strongly depended on the initial SOC content, e.g., sites with low SOC had more opportunity to increase their SOC than sites with high SOC. However, both these latter studies were conducted in wetter climates than that of the present study. Using data from 20 regions in the world, Minasny et al. [35] showed a tendency of a higher C sequestration potential on croplands with low initial SOC stock (≤ 30 t C / ha at 0–30 cm) compared to grasslands, which already have a high initial SOC stock, although a general decrease of the stock rate with time was also reported.

In addition, most of the samples in the present study were derived from the thermo-Mediterranean bioclimatic area [77], so these results can also reflect the latency of SOC variation in these areas, which even under soil abandonment showed limited increases in SOC when compared to other bioclimates in the same region [78].

Therefore, such an approach gave us the chance to show that there was no significant increase from 1994 to 2017 when compared to the estimated increase from 1994 to 2008; this was partly expected given that a SOC mean of the SOC94 extracted from the LEG-SOC94 was slightly higher than that of the complete LEG-SOC94 dataset. In few exceptions (3 samples out of 30) there was an increase in SOC concentration that can also be due to sediment redistribution and deposition following erosion from the sites at higher altitudes or step slopes [79–81], changes in tillage depth or soil compaction [82]. In addition, in this area soil respiration due to increasing temperatures [83] may have offset any potential increase in SOC, and the 23-year time span may have not been sufficient to detect a SOC change, as pointed by Saby et al. (2008). Soil management with ploughing and the increasing mean temperature [83] are not conducive to SOC sequestration [85, 86]. In particular, Goidts and van Wesemael [86] showed that ploughing may override the increase in SOC over time. Also, an increase in SOC in ploughed soils is hard to achieve unless high quantities of organic residues and N are provided [87]. These two latter conditions are very limited in Sicily due to the low crop yield, the low amount of residues returned to the soil and scarce fertilization. Davidson and Janssens [88] showed that slight increases in temperature and water availability ratio may contribute in reducing SOC. In the present work, this may have occurred and may be what can be seen when comparing the expected change from 1994 to 2008 to the measured change from 1994 to 2017. In addition, the ratio between water availability and temperature may be increasing in the area under study, despite the fact that no direct report is available [83].

The lack of increase in SOC concentration found here (from 1994 to 2017), compared to those estimated in the period from 1994 to 2008, could also be due to a SOC reduction from 2008 to 2017 which cannot be excluded using the present data. Such a reduction from 2008 to 2017 might possibly be connected with the decoupling of EU Common Agricultural Policy (CAP) payments regarding agriculture in 2005 (Regulation EEC 1782/2003). Before this year, wheat was the continuous primary crop for arable lands and after this point crop rotation with legumes or fallow land was encouraged by new regulations. Notably, continuous wheat has been shown to favour SOC accumulation when compared to a high percentage of wheat-legume rotations or wheat-fallow rotations, in the same or similar environments [51, 89, 90]. Lastly, differences between the previous estimation (1994 to 2008) and the present measurements (1994 to 2017) can also depend on the differences between direct and indirect measurement [91], or transient changes in cultivation history that may have influenced the previous estimates [54], the latter of which was discarded here by an ad-hoc sampling in continuously ploughed soils with field crops.

Variability and confidence intervals regarding SOC suggest that the estimated change in the previous work [56] could have been affected by outliers or errors in the measurements related to analytical methods used [92], especially when used for highly alkaline soils with scarce SOC [69, 93], such as in the present study. These issues may have produced an apparent pattern of SOC accumulation from 1994 to 2008 that was not detected between 1994 and 2017. Problems in the estimate due to the sampling strategy [10, 94] were excluded, since the selection method used here provided a dataset with similar statistical properties to the original SOC distribution, and thus allowed for a maximum reduction in sampling sites.

Mitigation strategies and international projects have contributed to a debate regarding SOC sequestration. These traits are increasingly being taken into account in EU subsidies to the agricultural sector [95, 96].

There were different ongoing discussions following the paper by Sommer and Bossio [33] and more recently after the Soil 4 × 1000 initiative [35], all of which are above all pivotal to a reliable estimation of SOC dynamics. Zomer et al. [34] modelled the estimated increase in SOC concentration and stocks at a global scale in croplands and found that an average increase of approximately + 26%, which is similar to the estimated value found from 1994 to 2008. However, the estimation of [34] may not be suitable for small scale assessment and mapping and the use of legacy information can be crucial to confirm these trends.

### Conclusions

In conclusion, the SOC change in arable lands estimated from 1994 to 2008 through models built with non-paired data in the study area [56] was not confirmed by the measurement using paired sites in the 1994–2017 timespan. The discrepancies between the present data compared to the previously published estimates may depend on various factors, including: (i) possible errors in 1994 measurements and 2008 estimates, (ii) changes in land use, (iii) soil erosion. However, SOC concentration reduction from 2008 to 2017 cannot be excluded.

This result has a direct implication for the SOC monitoring network in the mid-term (e.g., 15–25 years) and its implication in a C accounting system. Results also encourage to support legacy data measurements of soil properties by reliable information on the land use, land use changes and soil management practices. These latter aspects were taken into account here, although indirectly, by choosing sites with no change in the land use or soil management, and could be used to correct the effect of other environmental traits (e.g., rainfall, slope). Direct information regarding the variation in SOC concentration and SOC stock in topsoil and subsoil in areas that are prone to degradation are urgently needed to drive policy making.

A debate on subsidies should take into account the information from the present work to ensure that subsidies will foster the landscape and regional environmental sustainability and provision of ecosystem services and to minimize regional differences due to unpaired data collection and analysis.

Further works should aim at: (1) increasing the number of sites to be resampled which would be derived from both the legacy collections of 1994 and 2008, (2) gathering information regarding land use dynamics in paired sites, (3) quantifying the effect of soil erosion in the flow of SOC within and among catchments; (4) gathering information on the change of soil properties in homogeneous areas at varying the land use or soil and crop managements.

Further scopes include, but are not limited to, studying a) errors in the determination methodologies of SOC concentrations in soils from different climatic regimes, and especially aridity and carbonate concentrations; b) changes in bulk density with time, depth, and soil and crop management practices; and c) the influence of wildfire and arson.

## Data Availability

The legacy data that support the findings of this study were made available from [the Regional Bureau for Agriculture, Rural Development and Mediterranean Fishery, the Department of Agriculture, ARTA. Palermo]. The new data samples taken in 2017 can be obtained upon request. The legacy data are subjected to licensing; in this study they were used under an agreement.
